# Mechanistic insights into non-coding Y RNA processing

**DOI:** 10.1080/15476286.2022.2057725

**Published:** 2022-03-30

**Authors:** Martina Billmeier, Darrell Green, Adam E. Hall, Carly Turnbull, Archana Singh, Ping Xu, Simon Moxon, Tamas Dalmay

**Affiliations:** a School of Biological Sciences, University of East Anglia, Norwich Research Park, Norwich, UK; b Institute of Medical Microbiology and Hygiene, University of Regensburg, Regensburg, Germany; c Norwich Medical School, University of East Anglia, Norwich Research Park, Norwich, UK; d Horizon Discovery, Cambridge Research Park, Waterbeach, UK; e Department of Plant Sciences, University of Cambridge, Cambridge, UK; f Shanghai Engineering Research Center of Plant Germplasm Resource, College of Life Sciences, Shanghai Normal University, Shanghai, China

**Keywords:** Y RNA, cancer, apoptosis, non-coding RNA, small RNA

## Abstract

Y RNAs (84–112 nt) are non-coding RNAs transcribed by RNA polymerase III and are characterized by a distinctive secondary structure. Human Y RNAs interact with the autoimmune proteins SSB and RO60 that together form a ribonucleoprotein (RNP) complex termed RoRNP and Y RNAs also perform regulatory roles in DNA and RNA replication and stability, which has major implications for diseases including cancer. During cellular stress and apoptosis, Y RNAs are cleaved into 3’ and 5’ end fragments termed Y RNA-derived small RNAs (ysRNAs). Although some ysRNA functions in stress, apoptosis and cancer have been reported, their fundamental biogenesis has not been described. Here we report that 3’ end *RNY5* cleavage is structure dependent. In high throughput mutagenesis experiments, cleavage occurred between the 2^nd^ and 3^rd^ nt above a double stranded stem comprising high GC content. We demonstrate that an internal loop above stem S3 is critical for producing 3’ end ysRNAs (31 nt) with mutants resulting in longer or no ysRNAs. We show a UGGGU sequence motif at position 22 of *RNY5* is critical for producing 5’ end ysRNAs (22–25 nt). We show that intact RO60 is critical for ysRNA biogenesis. We conclude that ribonuclease L (RNASEL) contributes to Y RNA cleavage in mouse embryonic fibroblasts but is not the only endoribonuclease important in human cells.

## Introduction

The last decade has seen the discovery of several new classes of small RNA (sRNA) distinct from DICER/AGO2 dependent microRNAs (miRNAs). Unlike miRNAs many of these novel sRNAs are derived from pre-existing RNA polymerase III generated housekeeping RNAs but are not degradation products as they have been shown to have their own independent downstream functions [1,2]. Parent RNAs include small nucleolar RNA (snoRNA), small nuclear RNA (snRNA), ribosomal RNA (rRNA), transfer RNA (tRNA), vault RNA (vtRNA) and Y RNA [3]. The latter, Y RNAs, were initially discovered as components of the Y RNA/SSB/RO60 ribonucleoprotein (RNP) complex termed RoRNP observed in the sera of patients with the autoimmune disorders systemic lupus erythematosus (SLE) and Sjögren’s syndrome [4,5]. The pyrimidine-rich stem loops within Y RNA molecules contribute to RO60 function by guiding RO60 localization and its interactions with other proteins and RNAs, where RO60 regulates non-coding RNA quality control and stress responses [6]. Independent to RO60 associated mechanisms, Y RNAs are required for the initiation of chromosomal DNA replication in mammalian cells [7]. Y RNAs associate with unreplicated euchromatin in late G1 phase cell nuclei before the initiation of DNA replication [8]. Y RNAs interact and co-localize with the origin recognition complex (ORC) and the pre-replication complex protein CDT1 [8]. Y RNAs are then displaced from nascent and replicated DNA present in replication foci in a ‘catch and release’ mechanism [8].

Y RNA-derived sRNAs (ysRNAs) were first described in apoptotic cells where human Y RNAs *RNY1, RNY3, RNY4* and *RNY5* were rapidly and specifically cleaved in an apparent caspase-dependent manner generating shorter fragments between 22–25 nt plus larger fragments at 31 nt [9]. In this report it was suggested that nucleases that cleave Y RNAs were caspase-dependent since Y RNA cleavage was similar to that of small nuclear ribonucleoprotein U1 subunit 70 (*SNRNP70*) cleavage, which was caspase 3 (CASP3) dependent [9,10]. Experiments showed that the longer 31 nt fragments were associated with both SSB (previously termed La) and RO60 whereas the shorter 22–25 nt fragments only bound to RO60 [9]. These fragments were assumed to be Y RNA degradation products [11].

Sequencing experiments in precursor B cells derived from acute lymphoblastic leukaemia patients revealed several sRNA fragments mapping to the 3’ ends of *RNY3* and *RNY5* were highly enriched [12]. These sRNA fragments were mis-annotated as miRNAs and termed miR-1975 and miR-1979. These sRNAs were also observed in solid tumours but reporter assays showed that these transcripts lacked gene silencing activity confirming that these sRNAs were not actually miRNAs [13]. MiR-1975 and miR-1979 were removed from miRBase and re-annotated as a distinct class of sRNA derived from Y RNA [13].

We previously reported that ysRNA biogenesis is independent of the miRNA pathway [14]. We showed that ysRNAs were prevalent in normal and malignant cells and in stressed and unstressed cells [14]. *RNY5* derived ysRNAs had a similar abundance to miRNAs in normal cells [14]. Northern blots revealed that *RNY5* derived ysRNAs were significantly increased during apoptosis [14]. Pull down assays and anion exchange chromatography confirmed that *RNY5* derived ysRNAs were not present in an AGO2 complex [14].

Later experiments showed that ysRNAs were present in human plasma as circulating components of larger complexes ~100-300 kDa [15]. YsRNAs were upregulated in breast cancer patient samples suggesting a role for ysRNAs as cancer biomarkers [16]. A study investigating *RNY5* derived ysRNA transfer between primary and cancer cells showed that ysRNAs were highly abundant in extracellular vesicles (EVs) [17]. *RNY5* derived ysRNAs contained a 5’ end 8 nt motif (GUAGUGGG), which is the same motif required for initiation of DNA replication as well as a 3’ end 9 nt motif (CCCACUGCU) [18]. Normal cell exposure to ysRNA positive EVs derived from cancer cells resulted in rapid cell death [17]. Synthetic versions of 23 nt and 31 nt *RNY5* derived ysRNA triggered primary cell death in a dose dependent manner suggesting that cancer cell derived ysRNAs establish apoptosis in neighbouring cells [17].

Although ysRNAs were reported two decades ago and have been shown to induce cell signalling functions more recently, ysRNA biogenesis remains unclear. Knowledge on the specific mechanisms of ysRNA biogenesis will be critical for understanding their roles in disease processes and may facilitate future Y RNA-mediated clinical interventions.

## Results

### Intact RO60 binding site is essential for 3’ end ysRNA biogenesis

**Figure 1.**
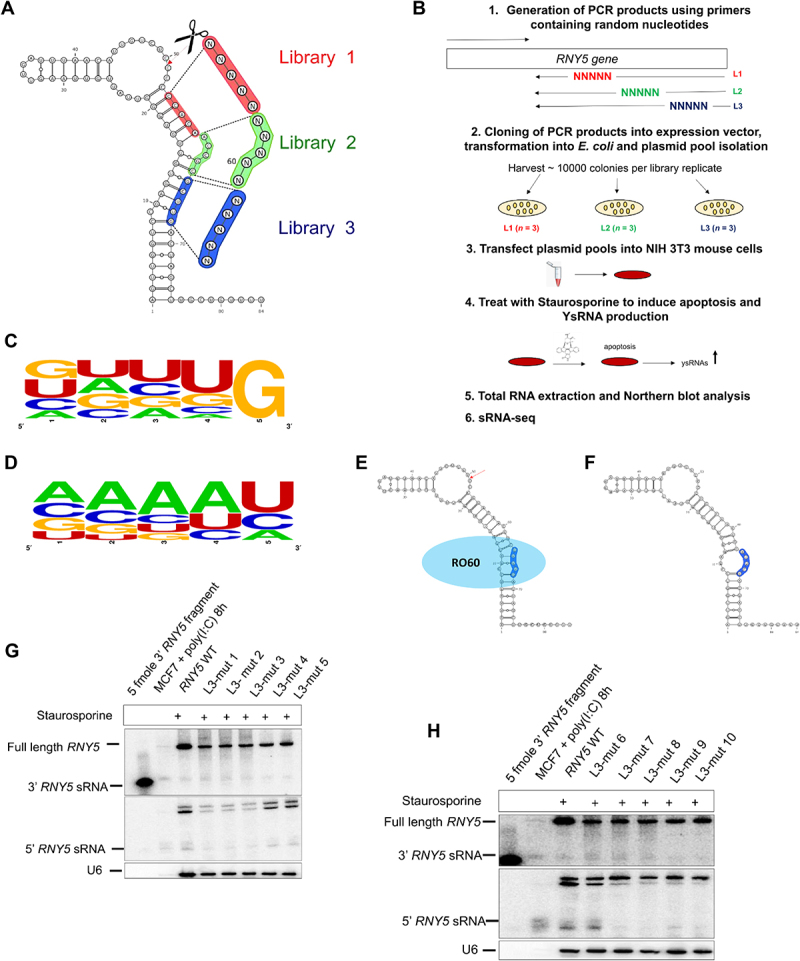
Intact RO60 binding site is essential for 3’ end ysRNA biogenesis. (A) Wild type human *RNY5* secondary structure (predicted) including the three mutant pool regions that were selected for 3’ end mutagenesis. In each of the mutant pools, L1 (red), L2 (green) and L3 (blue) near the 3’ end cleavage site (indicated between positions 49 and 50) 5 nt substitution mutations were introduced resulting in 1,024 possible combinations for each library. (B) Workflow of the 3’ end *RNY5* mutagenesis. The mutations for L1, L2 and L3 were introduced using primers containing random nucleotides. sRNA cDNA libraries were generated and sequenced. (C) Sequence logo analysis of most abundant L3 reads shows strong preference for G at the 5^th^ position. (D) Sequence logo analysis of missing mutant pool 3 reads shows absence of G at the 5^th^ position. (E) Predicted structure of a highly abundant mutant pool 3 sequence motif folds similar to wild type *RNY5* retaining an intact cytidine bulge for RO60 binding. The mutated region of L3 is depicted in dark blue whereas RO60 is shown in light blue binding to the cytidine bulge. (F) Predicted structure of a missing mutant pool 3 sequence motif indicates a change in the structure compared to wild type *RNY5*. The cytidine bulge and GC bp were no longer present, which affected the RO60 binding site plus full length mutant *RNY5* stability (mutated region of L3 is shown in blue). (G) Northern blot of most abundant L3 *RNY5* mutants show that these mutants generate ysRNAs at the same size as wild type *RNY5*. Total RNA extracted from human MCF7 cells treated with poly (I:C) and a synthetic 3’ end derived *RNY5* fragment of 31 nt were used as size markers for full length *RNY5* and wild type sized ysRNAs (throughout). Northern blot was probed with the 3’ and 5’ ysRNA probe. The blot was re-probed with U6 as a control. (H) Least abundant L3 *RNY5* mutants barely produce ysRNAs from the 3’ or 5’ end of *RNY5.*

We selected human *RNY5* as our model because *RNY5* expression can be more accurately assessed in murine cells as mice only express endogenous *Rny1* and *Rny3*. We cloned full length *RNY5* with 2.9 kb of 5’ flanking sequence plus 30 bp of downstream sequence into a pGEMT easy vector (Supplemental File 1A), which is required and sufficient to produce Y RNA transcripts from the plasmid. Our previous experiments using low throughput deletion/substitution mutagenesis within the *RNY5* stem loop structure showed that 3’ end cleavage correlated with structure rather than sequence (unpublished data) and that no Y RNA is detectable (neither full length or fragment) unless the cells are transfected with a Y RNA expressing plasmid or are treated with 1 M staurosporine (Supplementary File 1B). Here, to increase the number of *RNY5* mutants we designed a high throughput mutagenesis approach so that we could examine thousands of *RNY5* mutants in parallel. Three different 5 nt regions designated L1, L2 and L3 at the 3’ end were selected for mutagenesis. Each experimental region produced 1,024 possible nt combinations by randomly replacing all 5 nt with any of the four bases (Fig. 1A, B). The three mutant pools were transfected into mouse cells and followed by staurosporine treatment to induce apoptosis and ysRNA production (Fig. 1B). We performed sRNA-seq to profile ysRNAs from each mutant pool. *RNY5* mutants from L2 and L3 that were located furthest away from the 3’ end cleavage site and stem loop structure produced wild type sized ysRNAs (Supplemental File 1C). L1 mutants produced a characteristic double stranded stem comprising 5–6 nts plus an internal loop and cleavage occurred in all L1 mutants tested between the 2^nd^/3^rd^ nt above the stem resulting in different sized ysRNAs (up to 34 nt). We posited that this distinctive structural element was important for 3’ end ysRNA biogenesis. To confirm/check if an *RNY5* mutant was over or under represented in the plasmid pools, we sequenced the pools and confirmed that all expected 1,024 sequences were present (Supplemental File 1D).

To profile *RNY5* mutant levels in transfected cells, full length *RNY5* cDNA libraries were sequenced and mapped to all 1,024 possible sequences. The reads showed that several motifs in the L3 mutant pool were missing (Supplemental File 1E). We noted that L3 mutants were close in proximity to the RO60 binding site. We performed sequence logo analysis for the missing motifs and compared with the sequence logos of the 50 most abundant L3 motifs. We observed that the 5^th^ nt in L3 mutants showed an exclusive preference for G in the 50 most abundant mutants (Fig. 1C). This observation suggested that a GC base pair (bp) between *RNY5* positions 8 and 68 was necessary to retain the cytidine bulge, which was consistent with a previous finding [19]. U was the partially preferred 4^th^ nt (Fig. 1C). An explanation for this GU wobble is that this position requires some flexibility in order to produce a single nt bulge in the helix major groove so that RO60 can bind (Fig. 1E). This interpretation is consistent with the findings of two previous reports where removing the bulge by lower stem mutations resulted in reduced RO60 binding [20,21]. Sequence plus structure analysis (Fig. 1D) of the missing sequences revealed that those motifs alter the *RNY5* mutant structure in such a way that the structure lacks a cytidine bulge at position 8 and instead becomes an internal loop (Fig. 1F). In mutant sequences that were missing in the full length libraries, the GC bp at position 8 was absent and therefore the RO60 binding site was disrupted. These *RNY5* mutants scarcely produced ysRNAs (Fig. 1H) when compared to other L3 mutants where the RO60 binding site was intact (Figure 1G).

### 3’ end ysRNAs are Y RNA structure dependent

**Figure 2.**
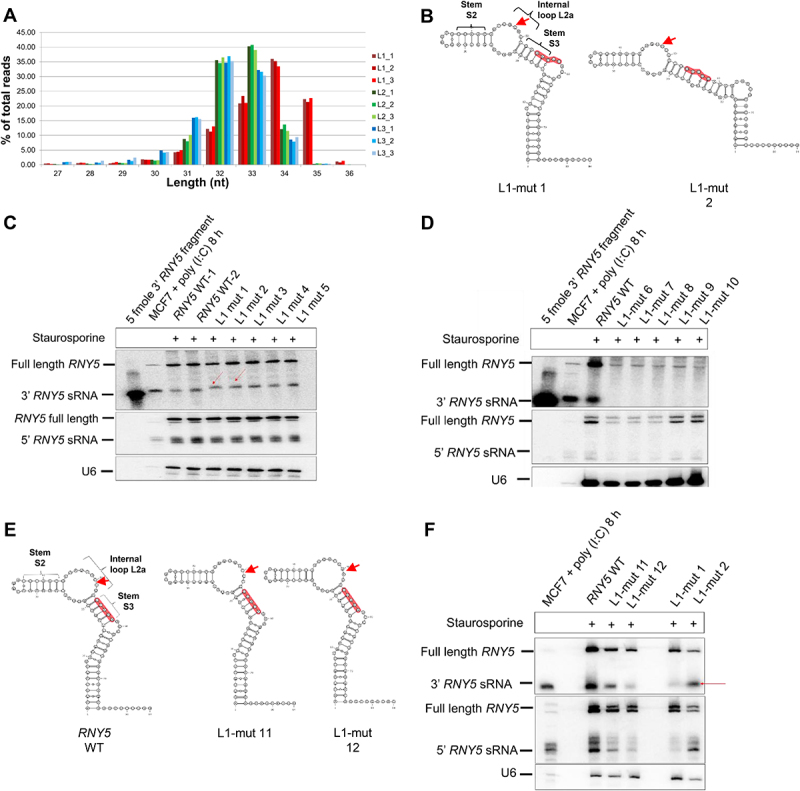
3’ end ysRNA production is Y RNA structure dependent. L1 mutants generate longer ysRNAs when compared to L2/L3 mutants. (A) Total read percentage for each cDNA library replicate of L1/L2/L3 mutant pools at each size ranging from 25–34 nts. L2/L3 mutant derived ysRNAs (green and blue) are mainly 32/33 nt. L1 mutant derived ysRNA reads (red) are longer with a length of 34/35 nt. (B) Predicted structures of the two most abundant L1 *RNY5* mutants generate longer ysRNA reads compared to wild type *RNY5*. (C) Most abundant L1 *RNY5* mutants generate longer ysRNAs compared to wild type *RNY5*. (D) Northern blot of the least abundant L1 *RNY5* mutants shows no ysRNAs. (E) L1 *RNY5* mutants with the same predicted structure as wild type generate wild type sized ysRNAs. Apart from the L1 *RNY5* mutants that fold the same way as wild type *RNY5* the structural features of wild type *RNY5* including the stem S, internal loop L2a and stem S3 close to the 3’ end cleavage site are shown. (F) L1 *RNY5* with the same structure than wild type *RNY5* produce wild type sized ysRNAs.

We generated sRNA libraries to investigate which 3’ end derived ysRNAs were most abundant and to examine which *RNY5* mutants were cleaved more/less efficiently. After adapter removal 57–75% of reads mapped to the mouse genome (RCm38/mm10) and the remaining reads mapped to mutant *RNY5*. Most L3 reads mapped to wild type *RNY5*. L2 and L3 mutant derived ysRNAs were 32/33 nt (Fig. 2A). L1 mutants produced longer ysRNAs at 34/35 nt (Fig. 2A). The start position of L2 and L3 reads indicated that cleavage occurred between U49/C50 and C50/C51 (Fig. 1A). This observation was consistent with our previous data obtained in MCF7 cells in which most of the 3’ end *RNY5* cleavage products were 31 nt with a cleavage site between C50/C51 (unpublished data). Given that L1 mutants produced longer ysRNAs when compared to wild type ysRNAs, we posited that the cleavage site had shifted. We ranked ysRNAs by abundance for the L1 mutants (Supplemental File 2A, B). Then we generated predicted structures for the most abundant *RNY5* mutants to see if the mutations affected RNA folding. We analysed the five most/least abundant ysRNAs derived from the L1 mutant pool by northern blot (Fig. 2C, D). The most abundant ysRNAs were those derived from the L1 mutants that most closely resembled the predicted wild type *RNY5* structure (Fig. 2B). The least abundant ysRNAs were those derived from the L1 mutants that were predicted to least resemble the wild type *RNY5* structure (Supplemental File 2D). L1 mutants that produced the most abundant ysRNAs generated longer fragments because the introduced mutations altered the structure in such a way that the Y RNA cleavage site had shifted. In these L1 mutants the internal loop L2a (Fig. 2B) shortened slightly so that Y RNA cleavage occurred 2/3 nt further upstream when compared to wild type *RNY5* but still 2 nt above the conserved stem (Fig. 2B). When the RNA structures of the five most/least abundant L1 mutants were analysed in more detail it was noted that in the L1 mutants that generated the most ysRNAs, stem S3 mainly consisted of 6 nt and had a high GC content (Fig. 2B; Supplemental File 2C). Sequencing also showed that the wild type *RNY5* stem S3 motif (CCACA, which was mutated in L1) was not the most abundant sequence amongst the longer 34/35 nt ysRNAs but was one of the most abundant sequences amongst the 32 nt ysRNAs. This finding was consistent with our model that the predicted *RNY5* structure and not the sequence was critical for 3’ end *RNY5* cleavage.

These data were supported by northern blots confirming that some L1 mutants generating abundant ysRNAs produced longer 3’ derived ysRNAs when compared to wild type ysRNAs (Fig. 2C). The least tolerated predicted structural changes did not produce 3’ end derived ysRNAs when compared to wild type *RNY5* (Fig. 2D; Supplemental File 2D). Two L1 mutants were predicted to fold similarly to wild type *RNY5* (Fig. 2E). Northern blot showed that these mutants generated wild type sized ysRNAs (Fig. 2F) supporting our hypothesis that Y RNA cleavage was structure dependent rather than sequence dependent. Y RNA cleavage occurred between the 2^nd^/3^rd^ nt above the conserved double stranded stem loop region S3 in the internal loop L2a.

### Internal loop 2a is required for 3’ end Y RNA cleavage

**Figure 3.**
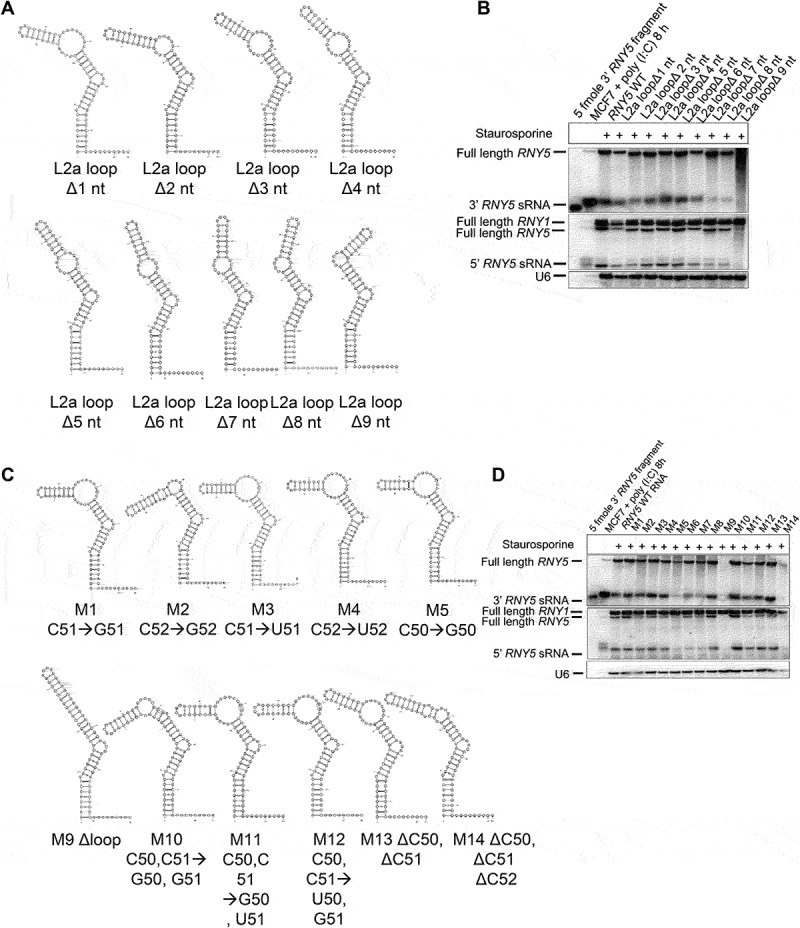
A bulge of 1 nt is required for 3’ end Y RNA cleavage. (A) All L2a loop mutants except for L2a Δ8 nt and L2a Δ9 nt were predicted to fold similarly to wild type *RNY5*. (B) Sequential deletion of internal loop L2a had a small effect on ysRNA production from either the 3’ or the 5’ end of *RNY5*. Except for L2a loop Δ3 nt to L2a loop Δ6 nt, mutants showed decreased ysRNA levels when compared to wild type *RNY5* derived ysRNAs. L2a loop Δ9 nt did not produce ysRNAs from neither the 3’ nor the 5’ end of *RNY5*. (C) Most of the *RNY5* substitution mutants except for M9 and M14 folded similarly to wild type *RNY5*. (D) Only M5 and M7 were less efficiently processed to ysRNAs when compared to wild type *RNY5.*

Some L1 mutants shortened the internal loop L2a in such a way that 3’ end *RNY5* cleavage happened further upstream but still 2 nt above stem S3 (Fig. 2B; Supplemental File 2C). We next investigated whether the internal loop length was important for 3’ end *RNY5* cleavage. We generated nine *RNY5* mutants termed L2a loop mutants, in which the loop structure was sequentially removed from the 5’ end of *RNY5*. Predicted structure analysis of L2a loop mutants showed that all of the deletion mutants except for L2a Δ8 nt and L2a Δ9 nt folded similarly to wild type *RNY5* (Fig. 3A). We transfected all of the L2a loop deletion mutants into 3T3 cells and treated the cells with staurosporine to induce ysRNA production. We compared ysRNA accumulation between each loop mutant by northern blot. These experiments showed that sequential deletion of internal loop L2a had a small effect on ysRNA production from both the 3’ and 5’ ends when compared to wild type *RNY5* derived ysRNAs (Fig. 3B). Except for the Δ3 nt to Δ6 nt mutants, the L2a loop mutants showed slightly decreased ysRNA levels when compared to wild type ysRNAs (Fig. 3B). L2a loop Δ9 nt did not produce ysRNAs from the 3’ or the 5’ end (Fig. 3B). This data plus the lack of clear full length *RNY5* signal suggested the L2a loop Δ9 nt mutant *RNY5* was unstable. These data showed that a 1 nt structural bulge element was sufficient for 3’ end *RNY5* cleavage. All L2a loop mutants were processed except for L2a loop Δ9 nt where the loop was completely removed, confirming that a bulge of at least 1 nt is critical and sufficient for 3’ end Y RNA cleavage.

### 3’ end Y RNA cleavage is sequence independent

We next sought to determine the importance of Y RNA sequence in addition to these predicted structural features. We previously detected in MCF7 cells that *RNY5* cleavage at the 3’ end mainly occurs between C50/C51 generating 32 nt long ysRNAs (unpublished data). Here we generated several *RNY5* mutants in which the cytosines at positions 50, 51 and 52 were substituted/deleted (Fig. 3C). Another hypothesis that we wished to examine was whether cytosine methylation could be involved in Y RNA cleavage as has been shown to occur in vtRNA cleavage [22]. Most of the *RNY5* substitution mutants except for M9 and M14 were predicted to fold similarly to the wild type (Fig. 3C). Nucleotides at positions 50, 51 and 52 did not appear to have any effect on Y RNA cleavage (Fig. 3D). All of the mutants except for M9 and M14 ΔLoopL2a were cleaved and generated ysRNAs (Fig. 3B, D). M5, M6 and M7 were less efficiently processed to ysRNAs when compared to wild type (Fig. 3D). Northern blot showed that full length *RNY5* M5 and M7 transcript levels were much lower when compared to other *RNY5* mutants, indicating that these mutants were less stable at the full length transcript level when compared to other mutants and less ysRNAs were generated (Fig. 3D). These data indicated that the cytosine residues at positions 50, 51 and 52 were not necessary for 3’ end *RNY5* cleavage. The M9 ΔLoopL2a mutant generated by making the sequence complementary to the sequence of loop L2b (Fig. 3C) showed no full length *RNY5* could be detected by northern blot (Fig. 3D). The M9 mutant altered the predicted structure in such a way that it became a double stranded RNA, which human cells degrade. When C50 was mutated to U or G (M6, M8) no effect on full length *RNY5* transcript level or Y RNA processing was observed when compared to the wild type (Fig. 3D). In M10, M11 and M12 positions 50 and 51 were mutated. Northern blot confirmed that the nt sequence at positions 50 or 51 did not affect Y RNA cleavage from the 3’ or the 5’ ends (Fig. 3D). YsRNAs were still generated at the same level when compared to the wild type even when two cytosines at positions 50 and 51 close to the 3’ end *RNY5* cleavage site were deleted. The predicted M13 mutant structure folded similarly to the *RNY5* wild type and contained 8 nt in the internal L2a loop whereas the wild type consisted of 9 nt (Fig. 3C). Deletion of three cytosines at positions 50, 51 and 52 in the M14 mutant altered the predicted structure in such a way that the single stranded internal L2a loop became shortened from 9 nt to 3 nt, which resulted in lower ysRNA accumulation when compared to the wild type (Fig. 3C, D). Shortening of the internal loop L2a decreased ysRNA generation when compared to the wild type. Y RNA cleavage still occurred when there was at least a 1 nt bulge. Mutagenesis of the cytosines indicated that the nucleotides at positions 50, 51 and 52 were not essential for 3’ end *RNY5* cleavage. Although not tested in detail this may suggest that m^5^C methylation does not play a role in 3’ end ysRNA generation.

### 5’ end Y RNA cleavage is UGGGU sequence dependent

**Figure 4.**
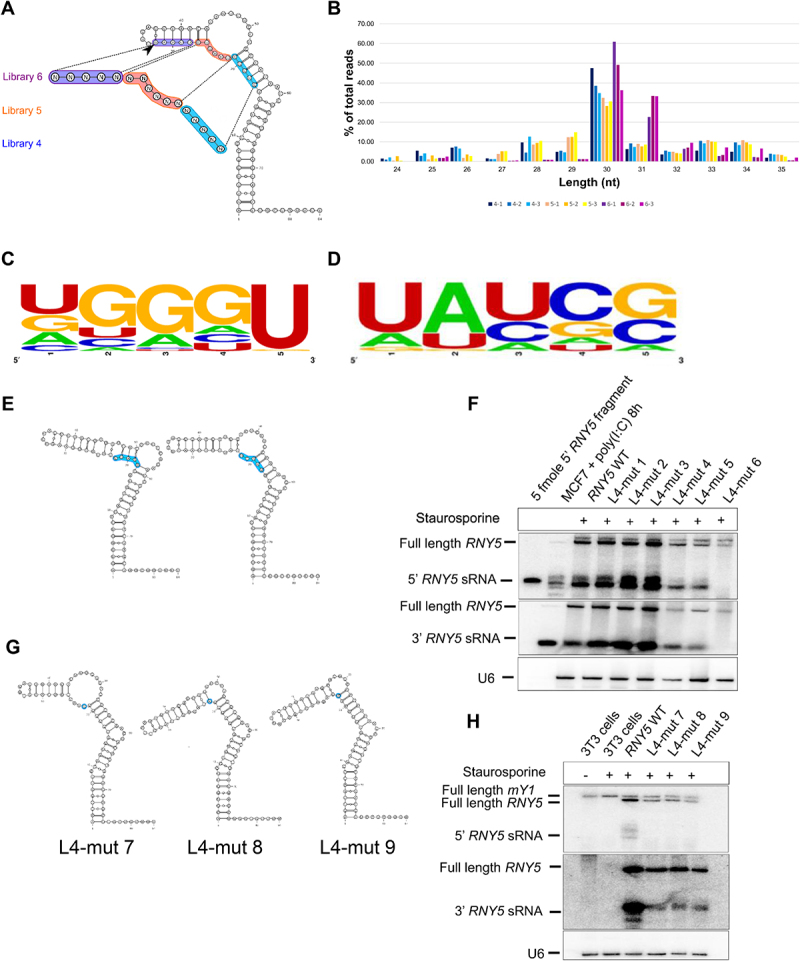
5’ end Y RNA cleavage is UGGGU sequence dependent. (A) Structure of wild type human *RNY5* including the three mutant pool regions that were selected for 5’ end mutagenesis analysis. In each of the regions of mutant pool L4 (blue), L5 (Orange) and L3 (purple) near the 5’ end cleavage site (indicated with an arrow between positions 32 and 33) 5 nt substitution mutations were introduced resulting in 1,024 possible combinations for each library. (B) L4/L5 mutants produced mainly 30 nt ysRNAs whereas L6 mutants produced mainly 30/31 nt ysRNAs. (C) Sequence logo analysis showed a strong selection for the wild type *RNY5* sequence motif UGGGU. (D) Sequence logo analysis showed that for all abundant L5 produced ysRNAs there was strong selection for the first three positions to be UAU. (E) Predicted L4 *RNY5* mutants fold similar to wild type *RNY5* and the two most abundant L4 *RNY5* mutants are shown as representatives. (F) L4 *RNY5* mutants generate wild type sized ysRNAs. (G) Predicted *RNY5* mutant structures with mutations at position 22. U was replaced by A, C and G. (H) Y RNA cleavage was affected at the 5’ end of *RNY5* if the U at position 22 of *RNY5* was mutated.

It was previously reported that a synthetic version of the 23 nt and 31 nt 5’ end *RNY5* fragments triggered apoptosis in a dose dependent manner [17]. There it was observed that the conserved 8 nt motif in the 5’ end derived *RNY5* fragment, notably important for DNA replication, is also required for apoptosis [17]. Similar to our 3’ end *RNY5* mutagenesis experiments we generated 5’ end mutagenesis libraries termed L4, L5 and L6 (Fig. 4A; Supplemental File 3A). We checked the distribution of all mutations and we observed all possible 1,024 mutants in each of the libraries (Supplemental File 3B). We profiled the accumulation of the full length mutant *RNY5* and this time we observed all possible 1,024 mutants in the full length libraries (Supplemental File 3C). We transfected *RNY5* mutant pools into 3T3 cells and treated with staurosporine to induce apoptosis and to generate ysRNAs. Size class distribution analysis showed that L4/L5 mutants produced mainly 30 nt ysRNAs whereas L6 mutants produced mainly 30/31 nt ysRNAs (Fig. 4B). L4 mutant sequence analysis showed a strong selection for the wild type *RNY5* sequence motif UGGGU (Fig. 4C). The mutated region of L4 is part of the 8 nt conserved sequence motif (GUUGUGGG) important for DNA replication and triggering apoptosis [17]. For L5 *RNY5* derived ysRNAs there was strong selection for UAU at the first three positions (Fig. 4D). Alignment of all human Y RNAs showed that the UAUU motif was conserved amongst human *RNY1, RNY4* and *RNY5* (Supplemental File 3D). Predicted structure analysis revealed that the most abundant L4 *RNY5* mutants folded in a similar way to wild type (Fig. 4E). This finding was consistent with the northern blot where the most abundant L4 *RNY5* mutants showed wild type sized ysRNAs except for L4-mut-6 where there are no ysRNAs (Fig. 4F).

We next investigated whether the wild type motif UGGGU is required for 5’ end ysRNA biogenesis. Sequence analysis showed a strong preference for U in the 5^th^ position (Fig. 4C). Sequence alignment of all human Y RNAs showed that U at the 1^st^ position, G at the 2^nd^ position and U at the 5^th^ position were conserved in all human Y RNAs (Supplemental File 3D). To check if the U at the 5^th^ position is essential, we replaced the U with A, C and G. We generated the *RNY5* mutants L4-mut 7 UGGGC, L4-mut 8 UGGGA and L4-mut 9 UGGGG and observed ysRNA accumulation (Fig. 4F). The UGGGC mutant was predicted to fold similarly to the wild type sequence (Fig. 4G). Northern blot showed that Y RNA cleavage was affected and inhibited at the 5’ end of *RNY5* in the L4 mutants when compared to wild type (Fig. 4H). These data infer that U at position 22 is essential for 5’ end *RNY5* cleavage.

### Conserved UUAU motif involved in 3’ and 5’ end cleavage

**Figure 5.**
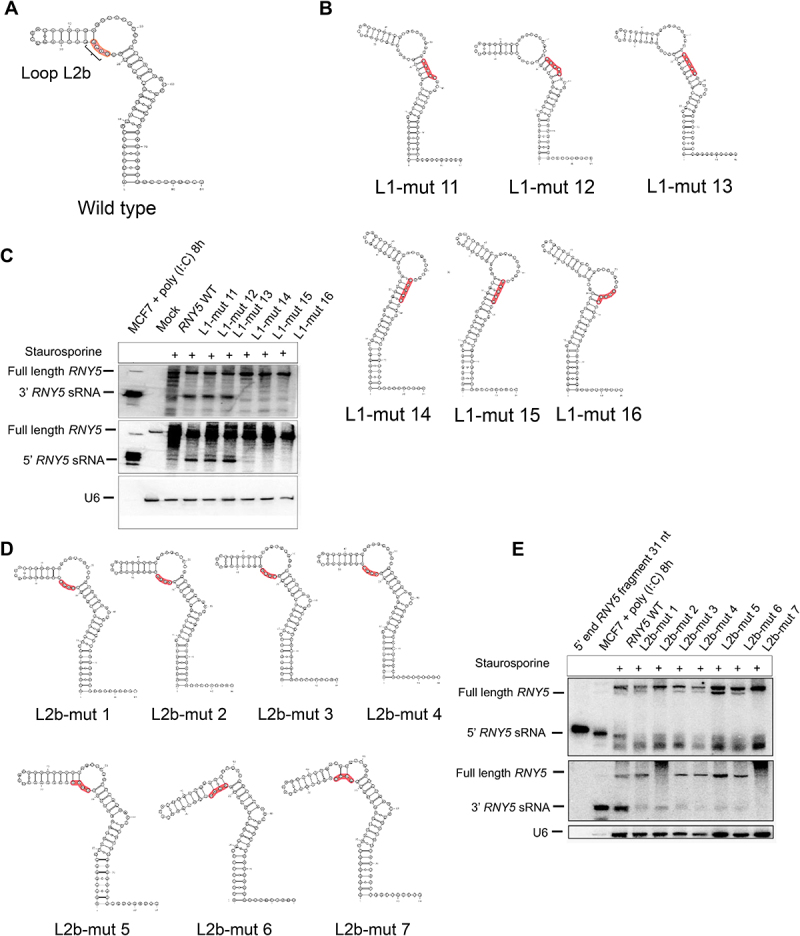
Conserved UUAU motif involved in 3’ and 5’ end cleavage. (A) Wild type *RNY5* structure with the structural feature loop L2b. The structural feature loop L2b at positions 23–26 is shown in Orange. (B) Predicted structures of *RNY5* L1 mutants with high or no ysRNA accumulation. *RNY5* L1 mutants that produce ysRNAs in a good amount are shown in the upper panel whereas the *RNY5* L1 mutants with no ysRNAs from the 3’ and 5’ end are shown in the lower panel. (C) *RNY5* L1 mutants with an intact internal loop L2b generate a good amount of ysRNAs whereas *RNY5* mutants with a shorter internal loop L2b produce less/no ysRNA from the 3’ nor the 5’ end of *RNY5*. (D) Predicted structures with mutated loop L2b with the same structure than wild type *RNY5* (upper panel) or different structure than wild type *RNY5*. (E) Mutations in loop L2b resulted in a lower accumulation of ysRNAs from the 3’ and 5’ end of *RNY5.*

As the UAUU motif at position 22–25 was conserved between *RNY1, RNY4* and *RNY5* (Supplemental File 3D) we analysed loop L2b as a structural feature in some *RNY5* mutants in more detail (Fig. 5A). For 3’ end *RNY5* mutagenesis, we observed that some L1 mutants were more efficiently cleaved than others. We looked at these predicted mutant structures and observed that L1 mutants that generated ysRNAs all folded in such a way that the loop 2b was intact in the mutants L1-mut 11 to 13 (Fig. 5B). Contrarily the *RNY5* mutants that were less efficiently cleaved showed that the loop L2b was shortened in L1 mutants L1-mut 14 to 16 (Fig. 5C). Northern blot confirmed that the L1 mutants with an intact loop 2b with the sequence UUAU were efficiently cleaved from the 3’ and 5’ end of *RNY5* whereas those mutants that resulted in a shortened loop 2b were not cleaved (Fig. 5C). To investigate if the sequence or structure of loop 2b was essential for Y RNA cleavage, several loop 2b mutants (L2b-mutants) tested by northern blot. L2b mutants that had the same predicted structure as wild type *RNY5* were compared to some L2b mutants that folded differently than wild type (Fig. 5D). Northern blot revealed that all of the L2b mutants resulted in a lower ysRNA accumulation from the *RNY5* 3’ and 5’ end when compared to wild type (Fig. 5E) indicating that the UUAU sequence motif was important for *RNY5* cleavage from both the 3’ and 5’ end.

### RO60 is critical for Y RNA cleavage

**Figure 6.**
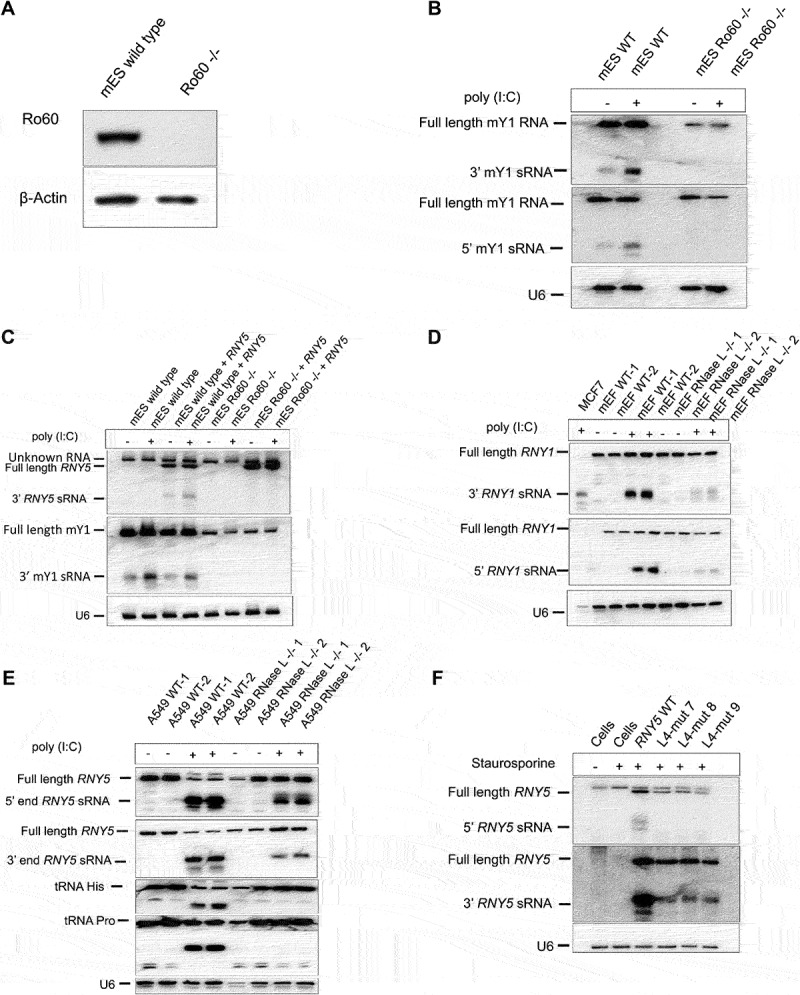
RO60 is critical for Y RNA cleavage. (A) Mouse embryonic stem cells lack Ro60 protein in a Ro60^−/−^ knock out cell line. (B) Ro60^−/−^ mES cells did not generate ysRNAs from the 3’ or 5’ end of *Rny1* (*mY1*) when cells were treated with poly (I:C) using the human *RNY1* probe. (C) RO60 is critical for *RNY5* cleavage at the 3’ end. The 5’ probe for *RNY5* appears to hybridize with an unknown RNA that is slightly longer than *RNY5*. (D) RNASEL contributes to Y RNA cleavage in mouse cells using wild type and RNASEL^−/−^ mouse fibroblast cells. (E) tRNA cleavage was abolished in RNASEL^−/−^ cells human cells upon poly (I:C) treatment confirming RNASEL removal in this mutant. RNASEL knock out did not affect ysRNA generation in human cells as much as it did in mouse cells. (F) *RNY5* substitution mutants in which the U of the RNASEL favoured UN^N motif was replaced by A, G or C. These mutants were transfected into 3T3 cells. The mutations introduced at position 49 did not have any effect on Y RNA expression or cleavage from the 3’ or 5’ end of *RNY5* in mouse cells.

We next asked whether RO60 was critical for Y RNA cleavage given that Y RNAs form a stable complex with SSB/RO60. Our earlier data inferred that Y RNA cleavage was impaired when the RO60 binding site was not intact. We generated sRNA libraries from wild type and Ro60^−/−^ mouse embryonic stem cells (mES) after poly (I:C) treatment. We confirmed mES genotype by western blot (Fig. 6A). Sequencing showed that upon poly (I:C) treatment sRNAs derived from *Rny1* and *Rny3* were markedly increased in the wild type whereas the Ro60^−/−^ mES cells were more or less unaffected plus northern blot confirmed that Ro60^−/−^ mES cells did not generate ysRNAs from *Rny1* (Fig. 6B). Wild type mES cells showed *Rny1* cleavage products from both the 3’ and 5’ end upon poly (I:C) treatment (Fig. 6B). These data showed that Ro60 is involved in the generation/protection of murine Y RNA fragments. We transfected wild type and Ro60^−/−^ mES cells with human *RNY5* and treated with poly (I:C) (Fig. 6C). Northern blot confirmed that Ro60 is critical for *RNY5* cleavage at the 3’ end as no ysRNA production could be observed in Ro60^−/−^ mES cells (Fig. 6C). We observed that full length *RNY5* was not decreased in Ro60^−/−^ mES cells but the 5’ probe for *RNY5* detected an unknown RNA slightly longer than *RNY5* not affected by STS or Ro60 (Fig. 6C). Ro60 did not have a role in other sRNA class biogenesis and was specific to Y RNAs (unpublished data).

### Ribonuclease L cleaves Y RNAs in mouse cells but not in human cells

Ribonuclease L (RNASEL) is a ribonuclease involved in the interferon-induced antiviral response. RNASEL inhibits viral replication and cell proliferation and induces apoptosis. RNASEL is activated by dsRNA and 2,-5,-oligoadenylate synthetase 1 (OAS1), which leads to global protein synthesis arrest [23,24]. A recent study showed that RNASEL cleaves tRNA and Y RNA when inducing protein synthesis arrest [25]. Here we show that RNASEL contributed to Y RNA cleavage in mouse cells using wild type and RNASEL^−/−^ mouse fibroblast cells (mEFs) (Fig. 6D). To perform the same test in human cells, we confirmed an RNASEL^−/−^ knock out (mutant A549 cells) by probing the blot with an oligonucleotide complementary to tRNAHis and tRNAPro (Fig. 6E). The northern blot showed that tRNA cleavage was abolished in RNASEL^−/−^ cells upon poly (I:C) treatment confirming that RNASEL was lacking (Fig. 6E). RNASEL knock out did not reduce ysRNA generation in human cells (Fig. 6E) as much as it did in mouse cells (Fig. 6D). We generated *RNY5* mutants in which the U of the favoured UN^N motif was replaced by A, G or C. These mutants were transfected into 3T3 cells. Northern blot showed that the mutations introduced at position 49 did not have any effect on Y RNA expression or Y RNA cleavage from the 3’ or 5’ end of *RNY5* (Fig. 6F). This data indicated that RNASEL might not be responsible for *RNY5* cleavage in human cells.

## Discussion

During cellular stress, Y RNAs are cleaved at the 3’ and 5’ ends producing ysRNAs. YsRNAs were first thought to be degradation products but were later shown to play key roles in apoptosis and other biological functions [17]. The question surrounding ysRNA biogenesis mechanisms has not been elucidated since other sRNAs are produced from their own loci or are processed by DICER or angiogenin (ANG) [26,27]. We previously showed that ysRNA biogenesis was DICER independent [14]. Here we show that ysRNAs are produced from the 3’ end of *RNY5* according to the presence of an internal loop above a conserved double stranded 5–6 nt stem S2 region. We show that ysRNAs are produced from the 5’ end according to the presence of a UGGGU sequence motif between positions 22–25 and others have shown it is these 5’ end derived fragments that have the most profound biological functions such as triggering apoptosis [17]. We identified the UUAU sequence motif at positions 22–25 to be critical for Y RNA cleavage. Finally, we showed that both 3’ and 5’ end *RNY5* derived ysRNAs require RO60 binding and that RNASEL is not essential for 3’ end RNY5 cleavage but contributes to murine Y RNA cleavage.

Our preliminary mutagenesis experiments indicated that 3’ end *RNY5* cleavage correlated with Y RNA structure (unpublished data). In these early experiments we found that a conserved double stranded stem region of 5–6 nt just below internal loop L2a was critical for 3’ end cleavage. These early studies were low throughput so here we developed a high throughput mutagenesis approach to examine thousands of *RNY5* mutants in parallel. Using this approach we showed that mutations in the most abundant *RNY5* L1 mutants changed the predicted structure in such a way that these mutants generated longer ysRNAs when compared to wild type. In all of these L1 mutants *RNY5* cleavage occurred between the 2^nd^/3^rd^ nt above the conserved base paired stem regardless of the sequence. As it was unknown which ribonuclease was involved in Y RNA cleavage our data suggested that components required for Y RNA cleavage might recognize structural features such as double stranded RNA and internal loops rather than a sequence. *RNY5* mutant structures were computationally predicted and were analysed by testing the expression levels of different *RNY5* mutants by northern blot. In these experiments it was possible to observe how internal loops and stem domains of *RNY5* mutants change.

For 5’ end ysRNAs we performed a high throughput mutagenesis approach similar to the 3’ end analysis. These *RNY5* mutants resulted in different sized ysRNAs ranging from 26–33 nt. An explanation for this size heterogeneity is that ribonucleases and/or proteins involved in Y RNA cleavage at the 5’ end are either not very specific or there are different ribonucleases generating different sized fragments. It can be speculated that these different sized fragments could perform different functions, for example, in extracellular vesicles or cell free. We showed that a UGGGU sequence motif in the internal loop L2b at positions 22–26 was essential for 5’ end *RNY5* cleavage. Sequence alignment of all human Y RNAs showed that this sequence motif is highly conserved, suggesting that it might be important for Y RNA and ysRNA biological function. Mutagenesis experiments of the U residue at position 22 revealed that U was critical for 5’ end *RNY5* cleavage and slightly affected cleavage at the 3’ end. The GUUGUGGG motif at position 14–21 that is close to the U residue at position 22 was previously shown to be involved in triggering apoptosis by cancer cells [17] and was essential for the DNA replication initiation [18].

To elucidate which ribonuclease/s were involved in Y RNA cleavage we investigated the role of RO60 and RNASEL. Y RNA cleavage via staurosporine and poly (I:C) treatment *in vitro* plus knockdown experiments using RNAi showed that RO60 was involved in ysRNA generation. Mutation of the RO60 binding site resulted in an inhibition of Y RNA cleavage. Sequencing plus northern blot showed that ysRNA generation was dependent on RO60 upon poly (I:C) treatment. RO60 did not affect the full length transcript level of transfected *RNY5* but Y RNA stability of mouse Y RNAs was decreased.

To investigate whether RNASEL was required for Y RNA cleavage, ysRNA production was tested in a mouse and a human RNASEL^−/−^ cell line. Northern blot of wild type and RNASEL^−/−^ mEF cells treated with poly (I:C) indicated that RNASEL was involved in Y RNA cleavage upon poly (I:C) treatment in mEF cells. In human RNASEL^−/−^ cells we observed that ysRNAs were still generated in the absence of RNASEL although ysRNA levels were clearly decreased. These results indicate that different ribonucleases and/or a different repertoire of proteins are involved in ysRNA production in mouse and human cells. Future experiments should aim to explore the RNases plus Y RNA interaction partners that are responsible for Y RNA cleavage in mouse and human cells. These experiments could be achieved by immunoprecipitation of biotinylated human and mouse Y RNAs followed by a pull down using streptavidin and mass spectrometry. With RNA therapies now appearing in the clinic, Y RNA manipulation may be of significant future clinical importance.

## Materials & methods

### Cell lines and cell culture

3T3 cells were cultured in DMEM (Gibco) with 10% FBS (Gibco), 1% L-Glutamine (Gibco) and 1% penicillin-streptomycin (Gibco). HeLa cells were cultured in EMEM (Sigma-Aldrich) with 10% FBS (FBS), 1% MEM non-essential amino acid solution (Gibco), 1% L-Glutamine and 1% penicillin-streptomycin (Gibco). MCF7 cells were cultured in low glucose DMEM + GlutaMAX (Gibco) with 10% FBS (Gibco), 1% MEM non-essential amino acid solution and 1% penicillin-streptomycin (Gibco). Wild type and Ro60^−/−^ mouse embryonic stem cells (mES) were provided by Professor Sandra Wolin (National Cancer Institute, USA). mES cells were cultured in knockout DMEM (Gibco), 15% ES cell FBS (Gibco), 1% L-Glutamine (Gibco), 1% MEM non-essential amino acid solution (Gibco), 100 U/ml ESGRO leukaemia inhibitory factor (Millipore) and 0.1 mM 2-Mercaptoethanol (Sigma-Aldrich). Wild type and RNASEL^−/−^ mouse embryonic fibroblast cells (mEF) were provided by Professor Robert Silverman (Case Western Reserve University, USA). RNASEL^−/−^ mEF cells were generated by the insertion of a neomycin resistance gene into the 5’ end of *RNASEL*. Wild type and RNASEL^−/−^ human cells were provided by Professor Susan Weiss (University of Pennsylvania, USA). Mouse and human wild type and RNASEL^−/−^ cells were cultured in RPMI 1640 medium (Gibco) with 1% L-Glutamine (Gibco) and 1% penicillin-streptomycin (Gibco). All cell lines were authenticated by STR profiling and were tested for *Mycoplasma* by PCR. We cultured cells at 5% CO_2_ and 37°C. We monitored cell viability by trypan blue staining.

### Transfection

We seeded 200,000 cells/well in six well plates (Sarstedt) with antibiotic-free media. After 24 h, we transfected cells with 20 ug of plasmid using Fugene 6 (Promega) according to manufacturer’s instructions. MCF7 and mES cells were transfected using Lipofectamine 2000 (Thermo Fisher Scientific) according to manufacturer’s instructions. For each transfection experiment appropriate controls including mock or empty vector were used.

### Poly (I:C) treatment

We prepared 2.5 mg/ml polyinosinic: polycytidylic acid potassium salt (poly (I:C)) (Sigma-Aldrich) in sterilized water. We treated cells with poly (I:C) at a final concentration of 10 μg/ml for 8 h before RNA extraction.

### Staurosporine treatment

Staurosporine (Cell Signalling Technology) was prepared as a stock in DMSO at 100 uM. We treated 3T3 cells at a final concentration of 1 uM for 4–8 h before RNA extraction.

### RNA extraction

Total RNA was extracted using TRI Reagent (Thermo Fisher Scientific). We measured total RNA concentration and integrity using the Nanodrop 8000 spectrophotometer (Thermo Fisher Scientific) plus visual assessment by 1.5% agarose gel electrophoresis.

### Northern blot

The gel was run at 110 V for 2–3 h in 0.5X TBE. We transferred RNA to a Hybond-NX (GE Healthcare) membrane using semidry transfer conditions at 250 mA for 45 m. We cross-linked sRNAs to the membrane by adding 5 mL cross-linking solution adjusted to pH 8 (12 mL water, 122.5 mL 12.5 M 1-methylimidazole, 10 mL 1 M hydrochloric acid and 0.373 g of 1-ethyl-3-(3-dimethylaminopropyl) carbodiimide) and incubated at 60°C for 1–2 h in saran wrap. For each sRNA we pre-hybridized the membrane with ultra-hyb-oligo buffer (Thermo Fisher Scientific) at 37°C for 1 h with rotation. We then incubated a mixture of 14 uL water, 2 uL 5X polynucleotide kinase forward buffer (New England Biolabs), 2 uL 10 mM DNA antisense oligonucleotide, 1 uL T4 polynucleotide kinase (New England Biolabs) and 3 uL [γ-^32^P]ATP (~1.1 MBq) at 37°C for 1 h. We incubated the membrane in this buffer rotating overnight at 37°C and then washed it three times in 0.2X SSC, 0.1% SDS before exposing on a phosphorimaging screen in a radioactive cassette (Fujifilm) followed by imaging on a Typhoon FLA 9000 (GE Healthcare). The membrane was re-probed using antisense DNA oligonucleotides (Sigma-Aldrich). We used *U6* as a loading control. Probes used against human Y RNA sequences were *RNY5* 3’ end wild type (5’-AGCTAGTCAAGCGCGGTTGTGGGGG-3’); *RNY5* 3’ end L1/L2 mutants (5’-AGCAAGCTAGTCAAGCGCGGT-3’); LNA 12 mer for L3 mutants (5’-*CAG*CAA*GCT*AG-3’); *RNY5* 5’ end wild type (5’-TAACCCACAACACTCGGACCAACT-3’); *RNY5* 5’ end L4/L5/L6 mutants (5’-CAACACTCGGACCAACT-3’); *RNY1* 3’ end (5’-AGACTAGTCAAGTGCAGTAGTGAGAA-3’); *RNY1* 5’ end (5’-TAACTCACTACCTTCGGACCAGCC-3’); tRNA-His (5’- CAGAGTACTAACCACTATACGATCACGGCC-3’) and tRNA-Pro (5’-CCGAGAATCATACCCCTAGACCAACGAGCC-3’); U6 (5’- GCTAATCTTCTCTGTATCGTTCC −3’).

### DNA constructs

We used a forward primer (5’-AATACTAGTGAAGATCCATGGAGGTACATC-3’) and reverse primer (5’-GTAAACGTTGTCTACTACTGTTATTAGTGC-3’) set to 64°C for 45s for primer annealing. PCR products were separated by 2% agarose gel electrophoresis, excised at the expected size and transferred to a 1.5 ml tube. We purified DNA using the Zymoclean gel DNA recovery kit (Zymo Research). DNA products were tailed and ligated into a pGEMT easy vector and transformed into DH5α *Escherichia coli* (*E. coli*) cells. Blue-white screening and colony PCR was performed to confirm the presence and size of the insert. Plasmid DNA purification was performed using the QIAprep Spin Miniprep Kit (Qiagen). Plasmid DNA samples were verified by direct sequencing.

### High throughput mutagenesis

Mutations were introduced using long primers containing random nt generating a pool of *RNY5* mutants for each of the six regions. The primers were synthesized in such a way that each of the bases was mixed equally with a proportion of 25% at each of the five mutated positions in order to ensure that each *RNY5* mutant would be equally distributed. The PCR for mutant *RNY5* pools L1-L6 was performed in three replicates. The resulting PCR products were A-tailed, ligated into pGEMT easy vector and transformed into *E. coli*. Statistical analysis revealed that for each mutant pool replicate a minimum of 5,000 colonies had to be harvested in order to be confident that each of the 1,024 possible *RNY5* mutants would be represented at least once with a probability of 99%. Colonies were harvested and plasmid DNA was purified using the HiSpeed midi prep kit (Qiagen). *RNY5* mutant pools were transfected into 3T3 cells. After 24 h, the cells were treated with staurosporine. After 8 h total RNA was extracted.

### sRNA-seq

cDNA libraries were constructed using 2 ug of RNA ligated to 3’ and 5’ high definition (HD) adapters as previously described [28,29]. We performed 50 bp single end sequencing on a HiSeq 2500 (Illumina).

### Bioinformatics

We converted FASTQ files to FASTA format and excluded reads containing unassigned nucleotides. HD signatures (four assigned nt at the ligating ends) plus the 3’ adapter were trimmed using perfect sequence matching to the first 8 nt of the 3’ HiSeq 2500 adapter (TGGAATTC). All reads were mapped to *RNY5* using PatMaN [30].

### Y RNA structures

We used RNAfold for *RNY5* predicted structure analysis [31] and Varna for *RNY5* predicted structure visualization [32].

## Supplementary Material

Supplemental Material

## Data Availability

All raw sequencing data for this study is available at Sequence Read Archive (www.ncbi.nlm.nih.gov/sra) under the accession PRJNA734120.
