# Supplementary material for: Mechanistic insights into non-coding Y RNA processing
Source: RNA Biol. 2022 Mar 30;19(1):468–80. doi: 10.1080/15476286.2022.2057725 (PMC8973356; doi:10.1080/15476286.2022.2057725)
Supplement: Supplemental Material [file KRNB_A_2057725_SM5391.zip › Supplemental_File_1.pptx]

## Slide 1
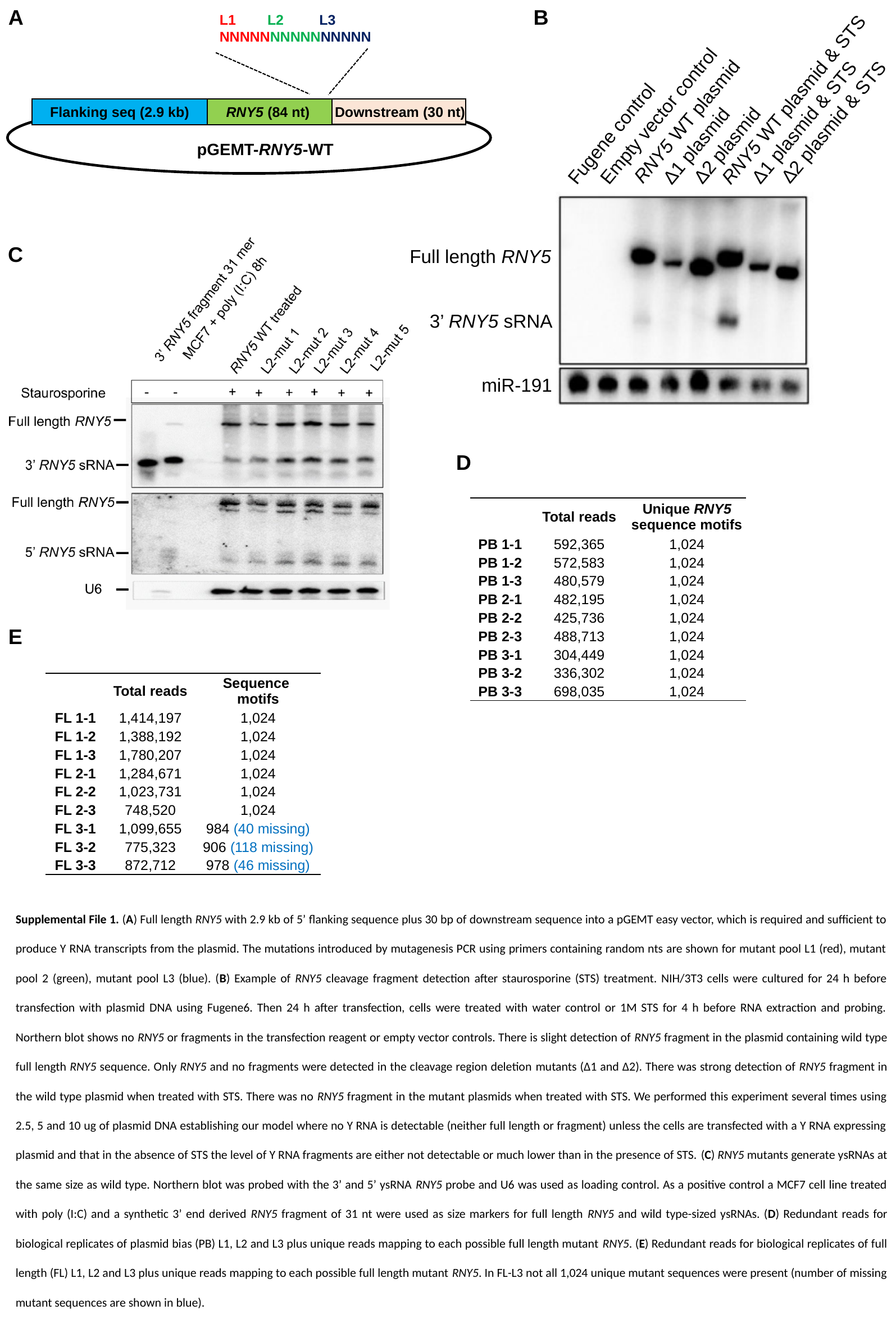

A
B
L1 L2 L3
NNNNNNNNNNNNNNN
Δ2 plasmid & STS
Δ1 plasmid & STS
RNY5 WT plasmid & STS
RNY5 WT plasmid
Δ2 plasmid
Δ1 plasmid
Empty vector control
RNY5 (84 nt)
Downstream (30 nt)
Flanking seq (2.9 kb)
Fugene control
pGEMT-RNY5-WT
C
Full length RNY5
3’ RNY5 sRNA
miR-191
D
| | Total reads | Unique RNY5 sequence motifs |
| --- | --- | --- |
| PB 1-1 | 592,365 | 1,024 |
| PB 1-2 | 572,583 | 1,024 |
| PB 1-3 | 480,579 | 1,024 |
| PB 2-1 | 482,195 | 1,024 |
| PB 2-2 | 425,736 | 1,024 |
| PB 2-3 | 488,713 | 1,024 |
| PB 3-1 | 304,449 | 1,024 |
| PB 3-2 | 336,302 | 1,024 |
| PB 3-3 | 698,035 | 1,024 |
E
| | Total reads | Sequence motifs |
| --- | --- | --- |
| FL 1-1 | 1,414,197 | 1,024 |
| FL 1-2 | 1,388,192 | 1,024 |
| FL 1-3 | 1,780,207 | 1,024 |
| FL 2-1 | 1,284,671 | 1,024 |
| FL 2-2 | 1,023,731 | 1,024 |
| FL 2-3 | 748,520 | 1,024 |
| FL 3-1 | 1,099,655 | 984 (40 missing) |
| FL 3-2 | 775,323 | 906 (118 missing) |
| FL 3-3 | 872,712 | 978 (46 missing) |
Supplemental File 1. (A) Full length RNY5 with 2.9 kb of 5’ flanking sequence plus 30 bp of downstream sequence into a pGEMT easy vector, which is required and sufficient to produce Y RNA transcripts from the plasmid. The mutations introduced by mutagenesis PCR using primers containing random nts are shown for mutant pool L1 (red), mutant pool 2 (green), mutant pool L3 (blue). (B) Example of RNY5 cleavage fragment detection after staurosporine (STS) treatment. NIH/3T3 cells were cultured for 24 h before transfection with plasmid DNA using Fugene6. Then 24 h after transfection, cells were treated with water control or 1M STS for 4 h before RNA extraction and probing. Northern blot shows no RNY5 or fragments in the transfection reagent or empty vector controls. There is slight detection of RNY5 fragment in the plasmid containing wild type full length RNY5 sequence. Only RNY5 and no fragments were detected in the cleavage region deletion mutants (Δ1 and Δ2). There was strong detection of RNY5 fragment in the wild type plasmid when treated with STS. There was no RNY5 fragment in the mutant plasmids when treated with STS. We performed this experiment several times using 2.5, 5 and 10 ug of plasmid DNA establishing our model where no Y RNA is detectable (neither full length or fragment) unless the cells are transfected with a Y RNA expressing plasmid and that in the absence of STS the level of Y RNA fragments are either not detectable or much lower than in the presence of STS. (C) RNY5 mutants generate ysRNAs at the same size as wild type. Northern blot was probed with the 3’ and 5’ ysRNA RNY5 probe and U6 was used as loading control. As a positive control a MCF7 cell line treated with poly (I:C) and a synthetic 3’ end derived RNY5 fragment of 31 nt were used as size markers for full length RNY5 and wild type-sized ysRNAs. (D) Redundant reads for biological replicates of plasmid bias (PB) L1, L2 and L3 plus unique reads mapping to each possible full length mutant RNY5. (E) Redundant reads for biological replicates of full length (FL) L1, L2 and L3 plus unique reads mapping to each possible full length mutant RNY5. In FL-L3 not all 1,024 unique mutant sequences were present (number of missing mutant sequences are shown in blue).
