# Supplementary material for: Mechanistic insights into non-coding Y RNA processing
Source: RNA Biol. 2022 Mar 30;19(1):468–80. doi: 10.1080/15476286.2022.2057725 (PMC8973356; doi:10.1080/15476286.2022.2057725)
Supplement: Supplemental Material [file KRNB_A_2057725_SM5391.zip › Supplemental_File_2.pptx]

## Slide 1
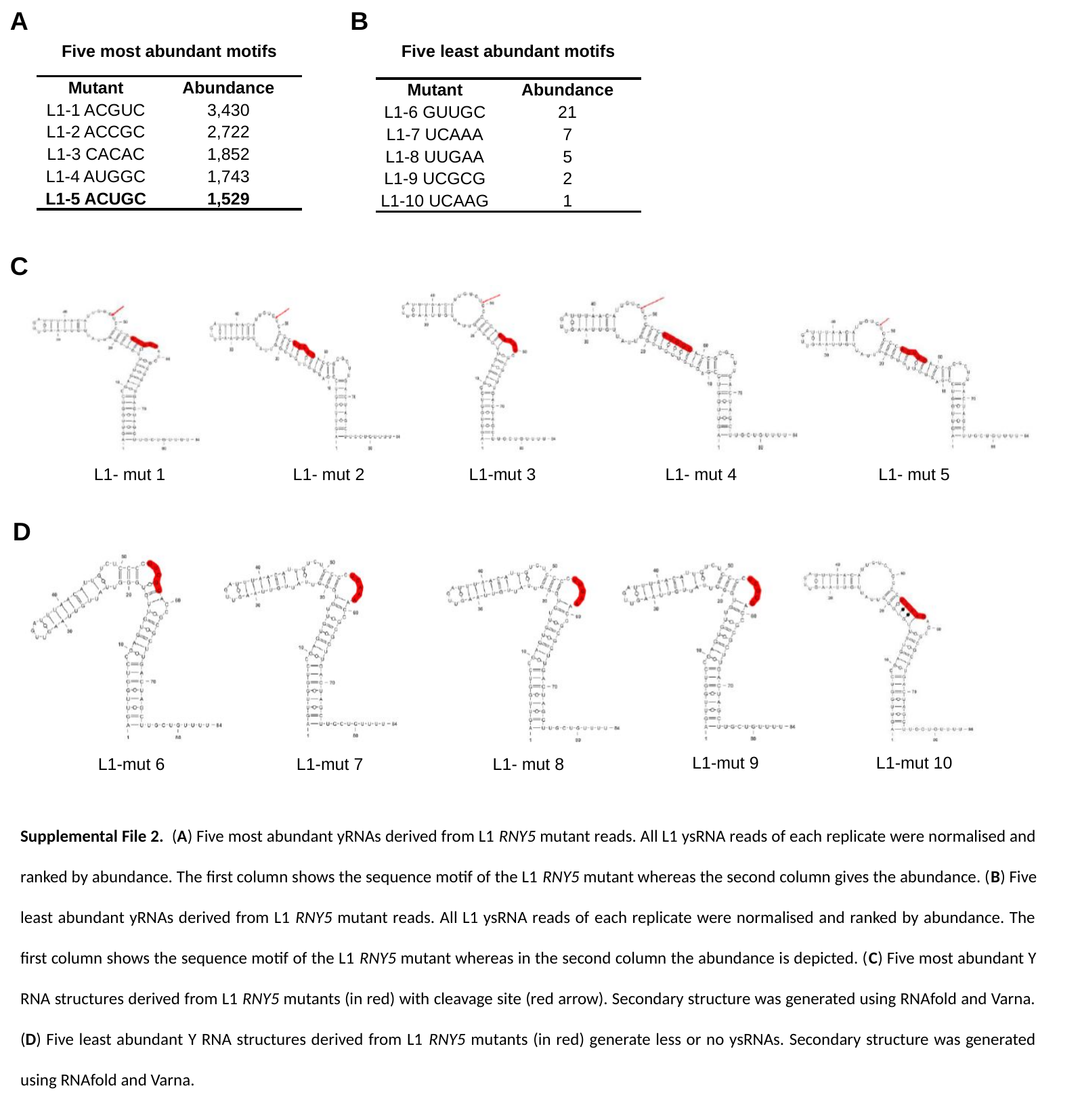

A
B
Five most abundant motifs
Five least abundant motifs
| Mutant | Abundance |
| --- | --- |
| L1-1 ACGUC | 3,430 |
| L1-2 ACCGC | 2,722 |
| L1-3 CACAC | 1,852 |
| L1-4 AUGGC | 1,743 |
| L1-5 ACUGC | 1,529 |
| Mutant | Abundance |
| --- | --- |
| L1-6 GUUGC | 21 |
| L1-7 UCAAA | 7 |
| L1-8 UUGAA | 5 |
| L1-9 UCGCG | 2 |
| L1-10 UCAAG | 1 |
C
L1- mut 5
L1-mut 3
L1- mut 4
L1- mut 2
L1- mut 1
L1-mut 10
L1-mut 9
L1- mut 8
L1-mut 7
L1-mut 6
D
Supplemental File 2. (A) Five most abundant yRNAs derived from L1 RNY5 mutant reads. All L1 ysRNA reads of each replicate were normalised and ranked by abundance. The first column shows the sequence motif of the L1 RNY5 mutant whereas the second column gives the abundance. (B) Five least abundant yRNAs derived from L1 RNY5 mutant reads. All L1 ysRNA reads of each replicate were normalised and ranked by abundance. The first column shows the sequence motif of the L1 RNY5 mutant whereas in the second column the abundance is depicted. (C) Five most abundant Y RNA structures derived from L1 RNY5 mutants (in red) with cleavage site (red arrow). Secondary structure was generated using RNAfold and Varna. (D) Five least abundant Y RNA structures derived from L1 RNY5 mutants (in red) generate less or no ysRNAs. Secondary structure was generated using RNAfold and Varna.
