# Supplementary material for: Mechanistic insights into non-coding Y RNA processing
Source: RNA Biol. 2022 Mar 30;19(1):468–80. doi: 10.1080/15476286.2022.2057725 (PMC8973356; doi:10.1080/15476286.2022.2057725)
Supplement: Supplemental Material [file KRNB_A_2057725_SM5391.zip › Supplemental_File_3.pptx]

## Slide 1
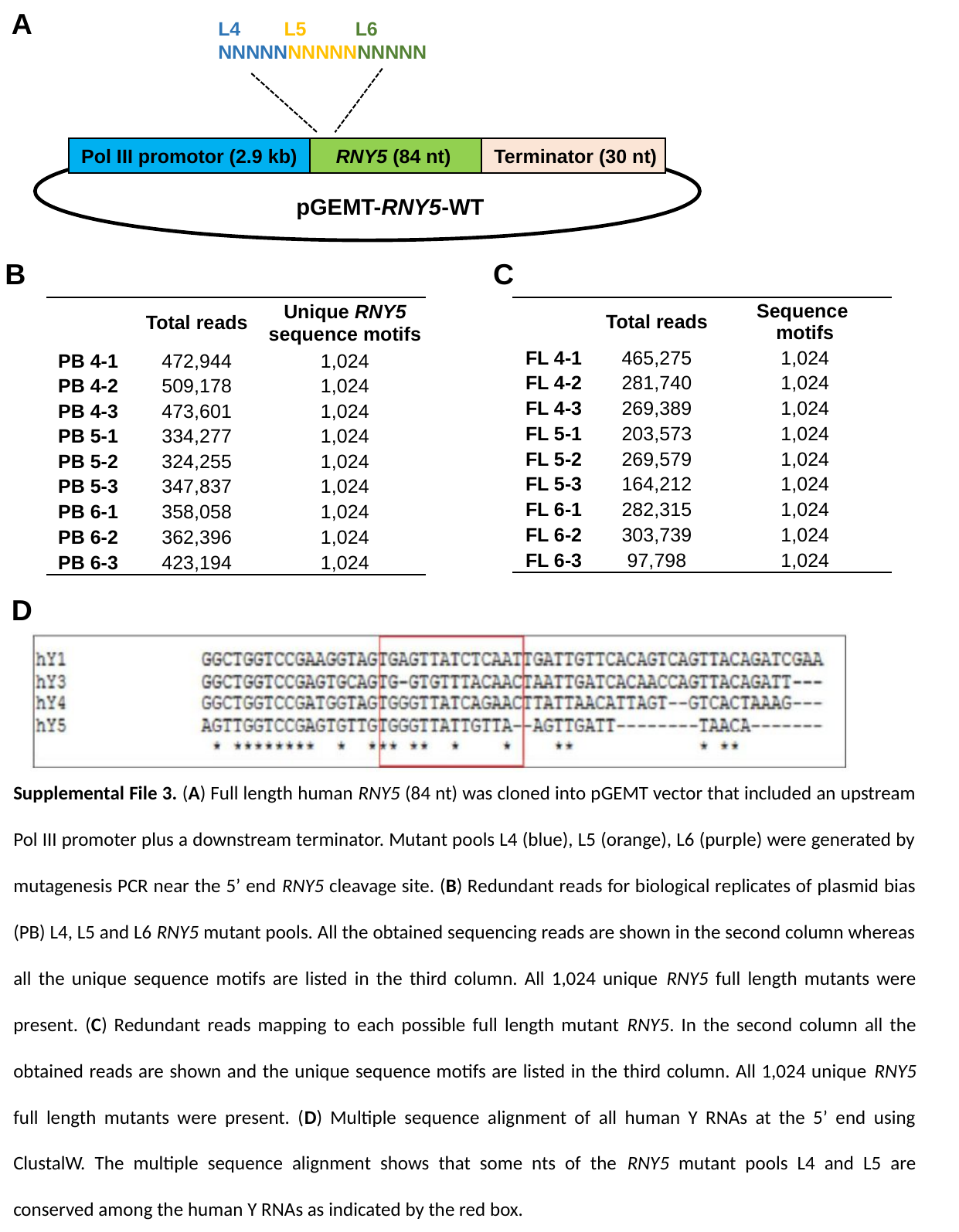

A
L4 L5 L6
NNNNNNNNNNNNNNN
RNY5 (84 nt)
Terminator (30 nt)
Pol III promotor (2.9 kb)
pGEMT-RNY5-WT
B
C
| | Total reads | Unique RNY5 sequence motifs |
| --- | --- | --- |
| PB 4-1 | 472,944 | 1,024 |
| PB 4-2 | 509,178 | 1,024 |
| PB 4-3 | 473,601 | 1,024 |
| PB 5-1 | 334,277 | 1,024 |
| PB 5-2 | 324,255 | 1,024 |
| PB 5-3 | 347,837 | 1,024 |
| PB 6-1 | 358,058 | 1,024 |
| PB 6-2 | 362,396 | 1,024 |
| PB 6-3 | 423,194 | 1,024 |
| | Total reads | Sequence motifs |
| --- | --- | --- |
| FL 4-1 | 465,275 | 1,024 |
| FL 4-2 | 281,740 | 1,024 |
| FL 4-3 | 269,389 | 1,024 |
| FL 5-1 | 203,573 | 1,024 |
| FL 5-2 | 269,579 | 1,024 |
| FL 5-3 | 164,212 | 1,024 |
| FL 6-1 | 282,315 | 1,024 |
| FL 6-2 | 303,739 | 1,024 |
| FL 6-3 | 97,798 | 1,024 |
D
Supplemental File 3. (A) Full length human RNY5 (84 nt) was cloned into pGEMT vector that included an upstream Pol III promoter plus a downstream terminator. Mutant pools L4 (blue), L5 (orange), L6 (purple) were generated by mutagenesis PCR near the 5’ end RNY5 cleavage site. (B) Redundant reads for biological replicates of plasmid bias (PB) L4, L5 and L6 RNY5 mutant pools. All the obtained sequencing reads are shown in the second column whereas all the unique sequence motifs are listed in the third column. All 1,024 unique RNY5 full length mutants were present. (C) Redundant reads mapping to each possible full length mutant RNY5. In the second column all the obtained reads are shown and the unique sequence motifs are listed in the third column. All 1,024 unique RNY5 full length mutants were present. (D) Multiple sequence alignment of all human Y RNAs at the 5’ end using ClustalW. The multiple sequence alignment shows that some nts of the RNY5 mutant pools L4 and L5 are conserved among the human Y RNAs as indicated by the red box.
